# Study on Personalized Recommendation Algorithm of Online Educational Resources Based on Knowledge Association

**DOI:** 10.1155/2022/2192459

**Published:** 2022-09-20

**Authors:** Ziqian Xu, Sheng Jiang

**Affiliations:** ^1^Huai'an Campus of Nanjing Forestry University, Nanjing, Jiangsu 210037, China; ^2^School of Mechatronics and Information, Wuxi Vocational Institute of Arts and Technology, Yixing 214206, China

## Abstract

In order to overcome the problems of low accuracy, low recommendation efficiency, and low user satisfaction of educational resources recommendation algorithm, this paper proposes a personalized recommendation algorithm for online educational resources based on knowledge association. Firstly, online education resources are collected according to association rules. Secondly, firefly algorithm is used to classify online education resources. Then, the vector space function is constructed to filter the classified online education resources. Finally, the correlation between knowledge points is calculated by knowledge association theory, and the knowledge with the highest user interest is selected as the target recommendation resource to realize the personalized recommendation of online education resources. The resource recommendation accuracy of this method can reach 97%, the recommendation time is less than 5.0 s, and users are more satisfied with it, indicating that its recommendation effect is good.

## 1. Introduction

At this stage, Internet technology and information processing technology are developing at a rapid speed, making people enter the big data era. Information resources provide many conveniences for people's life and work [1]. People can use information technology to establish databases, access and apply various data resources, and realize data resource sharing [2]. Although big data technology has brought many conveniences to people's lives, with the enrichment of data resources and the increase of user information content requirements, how to obtain resources that meet their own requirements has become the main demand at present. This issue has also become a research hotspot in the field of resource application [3]. Due to the development of online education technology, the demand for online education resources is gradually increasing, and the recommendation of online education resources has become the focus of relevant researchers [4].

Reference [5] proposed a collaborative filtering and recommendation method for online learning resources based on learner model. Based on education and teaching theory, as well as taking the resource utilization status in the teaching platform as a reference, a learning resource recommendation model was established, in which the dynamic and static characteristics of learners were integrated, and the collaborative filtering method was used to improve the model so as to realize resource recommendation. The experimental results show that this method has a wide coverage and can effectively recommend most resources, but the accuracy of the recommendation results is not high. Reference [6] proposed a personalized learning resource recommendation method based on three-dimensional feature collaborative domination, constructed the matching relationship between learning resources and learners, constructed the learning resource recommendation model according to the relationship between them, optimized the model parameters using three-dimensional feature collaborative matching method, and solved the model using particle swarm optimization algorithm to realize Learning Resource Recommendation. The experimental results show that this method has high recommendation efficiency. However, due to the lack of classification of resources, the adaptability between resources and users is not high, and there is a problem of low user satisfaction. Reference [7] proposed an online learning resource recommendation method based on multiobjective optimization strategy. Firstly, the objective function is established; that is, the learners' preference is the largest and the difficulty level is low. On the basis of meeting the above two objectives, the multiobjective particle swarm optimization algorithm is used to optimize and recommend online learning resources. The experimental results show that although this method can meet the needs of users it has the problem of low recommendation efficiency.

According to the above analysis, the existing methods not only have the problem of low accuracy of resource recommendation, but also have the problem of low recommendation efficiency, which affects the satisfaction of users. Therefore, a personalized recommendation algorithm for online education resources based on knowledge association is proposed. The specific research ideas of this paper are as follows.

Firstly, online education resource collection: Taking reliability, fault tolerance, and timeliness as standards, online education resources are collected according to association rules; Secondly, online education resources classification and online education resources filtering: Firefly algorithm is used to classify online education resources to improve the efficiency of resource recommendation and filter the online education resources by constructing vector space function classification.

Then, personalized recommendation of online educational resources: The correlation between knowledge points is calculated by knowledge association theory, and the knowledge with the highest user interest is selected as the target recommendation resource according to the measurement results so as to realize the personalized recommendation of online education resources.

Finally, the personalized recommendation effect of online education resources is verified by the accuracy of resource recommendation, the time of resource recommendation, and user satisfaction.

## 2. Personalized Recommendation Algorithm for Online Education Resources

### 2.1. Online Education Resource Collection

Because there are a large number of different types of educational resources in the network, in order to fully recommend online educational resources, first of all, we need to collect educational resources. This paper is mainly based on the theory of association rules. Association rules can not only reflect whether there is association between resources, but also describe the degree of association between resources [1, 8]. Understand the relationship between different educational resources, clarify the attributes of various types of resources, and then mine the resources to collect online educational resources.

Based on the theory of association rules, association rules are described by A⟶B. A and B represent itemsets, and there is no possibility of intersection between them; that is,

(1) $$\begin{array}{c} A\cap B=\emptyset \text{.} \end{array}$$

Considering the need to protect some resources, it is necessary to effectively ensure the confidentiality and integrity of online education resources. In order to effectively avoid malicious dissemination and disclosure of online education resources, educational resources are collected based on the following principles [9].

#### 2.1.1. Reliability

It is necessary to ensure that the online education resources collected through the network are accurate, true, and effective so as to effectively avoid misleading resource users.

#### 2.1.2. Fault Tolerance

If some online education resources in the network are maliciously spread or leaked, it is necessary to cut off the propagation path in time and ensure that the transmission paths of other resources in the network can work normally so as to ensure the security of all online education resources and the reliability of online education resources' downloading, uploading, and other operations. At the same time, try to reduce the operation cost generated in the process of online education resource leakage prevention, such as storage space, network energy consumption, and so on.

#### 2.1.3. Timeliness

Information resources in the network are changing rapidly, and users have high requirements for the efficiency of resource acquisition. Therefore, it is necessary to ensure a certain timeliness in resource acquisition so that users can obtain the most timely resource recommendation results.

**Figure 1:**
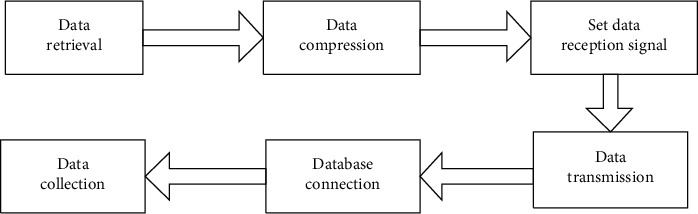
Flowchart of online education resource collection.

Based on the above principles, the specific process of online education resource collection is given as shown in Figure 1.

According to the resource collection process in Figure 1, the online education resources obtained are represented by set U, U={u_1_, u_2_,…u_n_}, where u_i_ represents the i education resource and n represents the number of resource types.

Set the data change interval in the online education resource set as [*α*, *β*], and then set a threshold value K to judge the attributes of online education resources. The expression of threshold value K is

(2) $$\begin{array}{c} K=\overset{N}{\underset{k=1}{\sum }}H_{k}+S_{k}\text{,} \end{array}$$

wherein H_k_ and S_k_, respectively, represent the educational resources corresponding to the input and output target knowledge points and N represents the number of resources.

In order to clarify the application frequency of different education resource types, attribute values are given to different education resource types, and the attribute weights of education resources in set U are expressed in the form of matrix as follows:

(3) $$\begin{array}{c} W_{k}=\left[\begin{array}{ccc} w_{k11} & w_{k12} & w_{k1n}\\ w_{k21} & w_{k22} & w_{k2n}\\ w_{kn1} & w_{kn2} & w_{knm} \end{array}\right]\text{,} \end{array}$$

where m represents the resource attribute type. The specific calculation formula of the attribute weight of educational resources is

(4) $$\begin{array}{c} W_{1}=\frac{\left(\max\;-W_{k}\right)}{\left(k-1\right)}\text{.} \end{array}$$

According to the attribute weights of different educational resources, the online educational resources are collected by comprehensively considering the threshold K; namely,

(5) $$\begin{array}{c} U\left(A,B\right)=\overset{n}{\underset{i=1}{\sum }}K_{i}{\left(A,B\right)}^{2}\text{,} \end{array}$$

where K_i_ represents resource coverage. To sum up, online education resources are collected.

### 2.2. Classification of Online Education Resources

In order to realize the omnidirectional recommendation of online education resources and further classify the resources, based on the resource collection results, this paper proposes a classification method of online education resources based on firefly algorithm so as to improve the efficiency of resource recommendation. Firefly algorithm is a heuristic optimization algorithm inspired by nature. The inspiration of the algorithm mainly comes from the flickering behavior of fireflies. The brightness of fireflies can be compared to a signal system to attract other fireflies and realize the clustering of fireflies [10, 11].

In order to successfully apply the firefly algorithm to the classification of resources, continuous variables must be converted into discrete variables. Therefore, the last two terms of formula (6) are converted into probability vectors by using logistic function. The logistic function is defined by the following formula:

(6) $$\begin{array}{c} P_{ij}=\frac{1}{1+e^{\vartheta_{ij}}}\text{,} \end{array}$$

where *P*_*ij*_ is the probability value of the *j*th dimension component of the feature vector represented by firefly *i*, wherein for *ϑ*_*ij*_ the function ensures that *P*_*ij*_ tends to 0 when *ϑ*_*ij*_ tends to positive infinity, and *P*_*ij*_ tends to 1 when *ϑ*_*ij*_ tends to negative infinity. The calculation formula of *ϑ*_*ij*_ is as follows:

(7) $$\begin{array}{c} \vartheta_{ij}=\beta_{0}e^{-\gamma r_{ij}^{2}}\left(x_{kj}-x_{ij}\right)+\alpha \left(\text{rand}-\frac{1}{2}\right)\text{.} \end{array}$$

The position update rule of the *i* firefly during the iteration of the algorithm is as follows:

(8) $$\begin{array}{c} x_{ij}^{t+1}=\left\{\begin{array}{cc} 1\text{,} & P_{ij}\geq \text{rand,}\\ 0\text{,} & P_{ij}<\text{rand.} \end{array}\right. \end{array}$$

**Figure 2:**
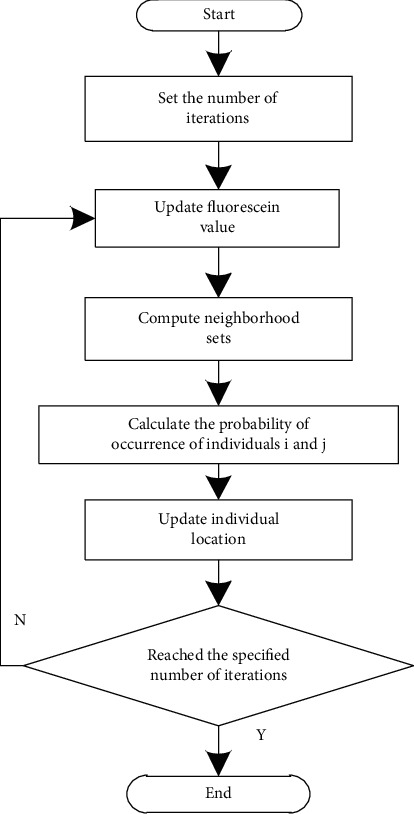
Classification flowchart of online education resources.

Based on the principle of the algorithm, online education resources are classified.  Figure 2 shows the detailed operation steps of online education resources classification based on the firefly algorithm.

According to Figure 2, the detailed operation steps of online education resource classification are as follows:

(1) Set the number of iterations to establish a solution space in which firefly individuals are randomly distributed.
(2) Update the fluorescein values of different firefly individuals.
(3) Calculate the neighborhood set of different firefly individuals, and the calculation formula is
  (9) $$\begin{array}{c} F^{2}=\exp\;{\left[\frac{\left({\left|f_{i,j}\right|}^{2}\right)}{\left({\left|f_{i+1,j+1}\right|}^{2}\right)}\right]}^{2}\times f\left[a,b\right]\text{,} \end{array}$$
where f_i,j_ represents the current neighborhood set; f_i+1,j+1_ represents the next neighborhood set; and a and b represent subsets in the neighborhood set.
(4) Calculate the probability that individuals i and j appear in the neighborhood set respectively:
  (10) $$\begin{array}{c} \left\{\begin{array}{c} P\left(i\right)=A_{ij}-A_{i},\\ P\left(j\right)=A_{ij}-A_{j}, \end{array}\right. \end{array}$$
where A_ij_ represents the overall probability of occurrence of individual i and individual j; A_i_ represents the probability of occurrence of individual i; and A_j_ represents the probability of occurrence of individual j.
(5) Calculate the probability that the target individual is selected, move the selected individual as the target, and update the position of the individual at the same time.
(6) Judge whether the algorithm has reached the specified number of iterations, and if so, output the final result; that is, realize the classification of online education resources. If not, skip to step (2).

According to the above analysis, after fluorescein update and calculation of individual occurrence probability, we can obtain the probability of different education resources' appearance, will be able to distinguish between education resources together, form the same feature set, in accordance with the principle of the set U all education resources classification processing, have access to education resources of different feature space vector, and realize the online education resources classification.

### 2.3. Online Education Resource Filtering

Based on the classification results of online education resources, further filter the resources. The purpose of this step is to avoid the impact of interference data on the effect of resource recommendation. In a broad sense, interference data refers to the data that does not meet the needs of users, repeated or wrong in the online education resource set. Recommending these data to users will not only affect the application effect of users on resources, but also affect the probability of useful resources being retrieved and recommended. Therefore, it is necessary to filter these resources [12]. This paper proposes an online education resource filtering method based on vector space function. Filtering resources using vector space function is mainly divided into two stages: training and filtering [13]. The training phase is to train the resource classification results to form a resource filter template. In the filtering stage, a filter board is mainly formed. The filter board is adjusted according to the user's feedback information to realize resource filtering.

According to the above analysis, firstly, the resource classification results obtained in Section 2.2 are trained to preliminarily screen out the invalid resources, and then the invalid resources are further screened out by taking the user's interest as a reference. Finally, the filtering and deletion process is realized to ensure the efficiency of educational resource recommendation. The educational resources in the network are not only diverse in content, but also constantly updated and iterated. It is very difficult to filter these resources. However, in order to recommend more comprehensive and accurate educational resources for users, it is necessary to preliminarily screen educational resources according to relevant standards.

Traditional filtering methods mainly reserve relevant resources according to users' interest points, while vector space functions provide users with resources with high interest and also remove the resources that users have no intention of establish a space vector model S(*μ*), whose expression is

(11) $$\begin{array}{c} S\left(\mu \right)=d_{a}+d_{h}+d_{g}+d_{e}+d_{v}\text{.} \end{array}$$

Among them, d_*a*_ represents bad resources; d_*h*_ represents unbalanced resources; d_*g*_ represents duplicate resources; d_*e*_ represents special resources; and d_*v*_ represents effective resources.

Train the constructed spatial vector model: define ∆T as the time complexity, assuming that the model contains M nonvalid resources, form them into a document, and traverse the document to obtain the traversed document expression:

(12) $$\begin{array}{c} S^{\prime }\left(\mu \right)=\frac{\sqrt{\overset{n}{\underset{k=1}{\sum }}∆T{\left(e_{i}-e_{k}\right)}^{2}\times {\left(g_{i}-g_{k}\right)}^{2}}}{M}\text{.} \end{array}$$

Among them, e_i_ and e_k_, respectively, represent the state vector of valid resources and nonvalid resources in the document; g_i_ and g_k_, respectively, represent the real distribution state of valid resources and nonvalid resources in the document.

Then, the goodness of fit of the distribution of ineffective resources in the document can be expressed as

(13) $$\begin{array}{c} F_{gk}=\omega_{n}^{2}\times \left(e_{k}+g_{k}\right)\text{,} \end{array}$$

where *ω*_n_^2^ represents the sparsity of resources in the document. Under normal circumstances, as the F_gk_ value continues to increase, the filtering precision of educational resources also increases.

### 2.4. Personalized Recommendation of Online Educational Resources

The learner knowledge correlation function is constructed, and the learner knowledge correlation function is applied to the recommendation technology of basic content, and the content recommendation algorithm based on knowledge correlation is proposed. In this recommendation algorithm, the problem of learners' cold start is solved by considering the knowledge attribute information of learners. The knowledge correlation function is used to predict and modify the learning path of learners to solve the problem that the continuity and systematization of learning cannot be guaranteed. By introducing learning style, the problem of single interest in content recommendation is solved. Finally, solve the problem of knowledge trek and theme drift in the process of learning and improve learning interest and learning efficiency.

According to the filtering results of online education resources, the personalized recommendation algorithm of resources is designed. This paper proposes a personalized recommendation algorithm of online education resources based on knowledge association. Knowledge association can be divided into two types: the association between knowledge points and the association between learning resources and knowledge points. The following two knowledge association modes are specifically analyzed.

#### 2.4.1. Correlation between Knowledge Points

The correlation between knowledge points means that the knowledge points of each subject are the basis of subject teaching. Although these knowledge points are divided in teaching research and practice, the knowledge points after the division are still related. This relationship can be divided into dependencies, siblings, and parent-child relationships.

#### 2.4.2. Association between Learning Resources and Knowledge Points

At this stage, we mainly extract keywords from learning resources through semantic association technology and then calculate the association between keyword vectors and knowledge points so as to obtain the correlation between them.

The specific construction and updating steps of the learner knowledge resource association model are as follows:

 
*Step 1*. Obtain the domain knowledge attribute of the learner from the learner's personal information table, such as discipline, grade, major, and so on, and then obtain the root knowledge point set *S*_*K*1_ associated with the domain knowledge of the learner according to the association relationship between the knowledge in the knowledge base.
 
*Step 2*. Obtain the learner's learning behavior data from the learner's learning behavior information table and get the resource set *S*_*R*_ that the learner has learned after cleaning and filtering. Further, according to the association relationship between knowledge points and resources, the knowledge point set *S*_*K*2_ associated with *S*_*R*_ and the corresponding association weight *W*_*i*_ are obtained.
 
*Step 3*. After obtaining the knowledge point sets *S*_*K*1_ and *S*_*K*2_, calculate the correlation weight between the knowledge point and the learner, wherein, if the knowledge point is not learned by the learner, the degree of association between the learner and the knowledge point is 0. On the contrary, the weight of the knowledge points and the learners is obtained by averaging the sum of the resources and the weights of the knowledge points.
 
*Step 4*. The updating of the association model of learners' knowledge resources mainly includes two situations: the change of learners' knowledge attribute information and the learners' learning of new learning resources. When these two situations occur, the above three steps are executed to obtain the latest knowledge set and association weight associated with the learner.

Based on the above theory, in order to realize the personalized recommendation of educational resources, the habit preference of learners is obtained from the needs of learners, the learning path of learners is mined, and the personalized recommendation of educational resources is realized in combination with knowledge association [14, 15].

First, establish a knowledge association model, select knowledge points Q_1_ and Q_2_ in the model, and calculate the weight of the two. The calculation formula is

(14) $$\begin{array}{c} \left\{\begin{array}{c} W\left(Q_{1}\right)=\frac{\delta_{1}\left(I-I_{k}\right)}{m_{k}}\text{,}\\ W\left(Q_{2}\right)=\frac{\delta_{2}\left(I-I_{k}\right)}{m_{k}}\text{.} \end{array}\right. \end{array}$$

Among them, *δ*_1_ and *δ*_2_ represent the set of knowledge points; I represents the parent knowledge point; I_k_ represents the child knowledge point; and m_k_ represents the sum of the associated weights of the knowledge points.

The knowledge point set composed of knowledge points Q_1_ and Q_2_ is obtained by formula (8), and the similarity between them is calculated by formula (8):

(15) $$\begin{array}{c} S\left(Q_{1},Q_{2}\right)=\frac{1-\left(f_{S}+f_{G}\right)}{f_{S}}\text{.} \end{array}$$

Among them, f_S_ represents the similarity relationship between resource attributes; f_G_ represents the similarity relationship between resource ontology.

Next, find the knowledge points related to learners' interest, measure the similarity between the knowledge points, and select the knowledge with the highest user interest as the target recommendation resource according to the measurement results. The measurement is mainly realized by calculating the cosine similarity between knowledge points:

(16) $$\begin{array}{c} S\left(Q_{1},Q_{2}\right)=\cos\left[\frac{Q_{1}.Q_{2}}{\left|Q_{1}\right|.\left|Q_{2}\right|}\right]\text{.} \end{array}$$

**Figure 3:**
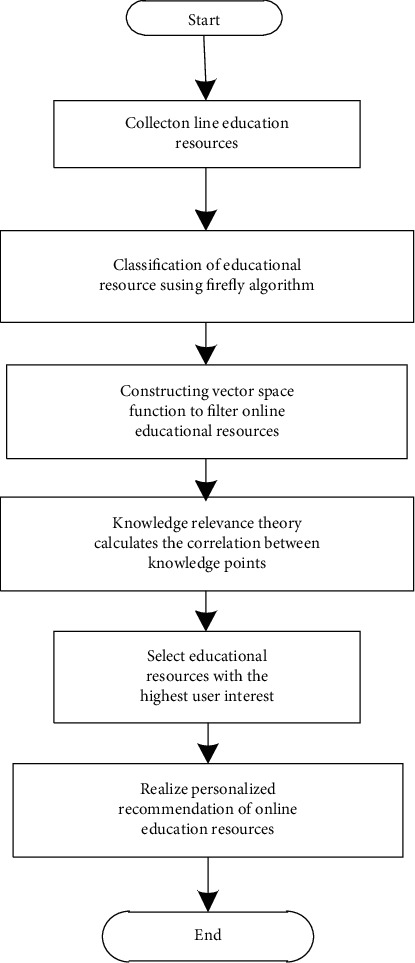
Personalized recommendation process of online educational resources.

Using formula (16) to get the similarity, recommend online education resources to learners, form a list of interest degrees, arrange the interest degrees in descending order, and then recommend education resources to learners in turn according to the arrangement results, and finally realize the personalized recommendation of online education resources. The personalized recommendation process of specific online education resources is shown in Figure 3.

Analysis of Figure 3 shows that online educational resources are collected according to the association rules, the firefly algorithm is used to classify the online educational resources, the classified online educational resources are filtered, the correlation between knowledge points is calculated by the knowledge association theory, and the user interest degree is selected. The highest knowledge is used as the target recommendation resource, and the personalized recommendation of online educational resources is completed.

## 3. Experimental Analysis

### 3.1. Experimental Design

There are two datasets used in this experiment:

1. *IntAddSub (Integer Addition and Subtraction) Dataset*. The dataset comes from an online learning platform and collects the real data of mathematics learning in grade 3 of a senior high school. The dataset mainly involves the function solving content in the senior high school mathematics of the people's education press, including 13 knowledge points. A total of 753 learners and 1056 exercises are collected. The time span is from February 23, 2021, to June 24, 2021. The data truly reflect the learning situation of learners. Personalized recommendation algorithm of online education resources based on knowledge association, which can personalized recommend online education resources to target learners
2. *FrcSub (Fraction Subtraction) Dataset*. This dataset is a public dataset. FrcSub collects exercise data on equation solving problems, which is often used by cognitive diagnostic models to validate the model's performance. The FrcSub dataset contains two parts of data: students' exercise scores and the relationship between exercise and knowledge points. It collects the score data of 536 learners on 20 exercises. Among them, 1 means that the exercise is answered correctly, and 0 means that the exercise is answered incorrectly. The relationship with knowledge points includes the investigation of 8 knowledge points in 20 exercises, among which, if the exercise examines the knowledge point, it is represented by 1; otherwise, it is represented by 0. The descriptive statistics of the two datasets are shown in Table 1.

On the basis of theoretical research, design experiments to verify the application performance of this method. Reference [5] method and reference [6] method are used as comparison methods to compare with this method. The specific experimental indicators are recommendation accuracy, recommendation efficiency, and user satisfaction. The data used in the experiment comes from the SQL Server database, in which the application statistics of online education of students in a university are extracted, including student resource retrieval data, resource download data, and resource submission data; based on the above data, the experimental research is carried out.

In this paper, recommendation accuracy, resource recommendation efficiency, and user satisfaction are selected as experimental evaluation indicators. The higher the calculation result, the better. On the contrary, the lower the calculation result, the worse.

### 3.2. Recommendation Effect Verification

#### 3.2.1. Comparison of Online Educational Resources Recommendation Accuracy

**Figure 4:**
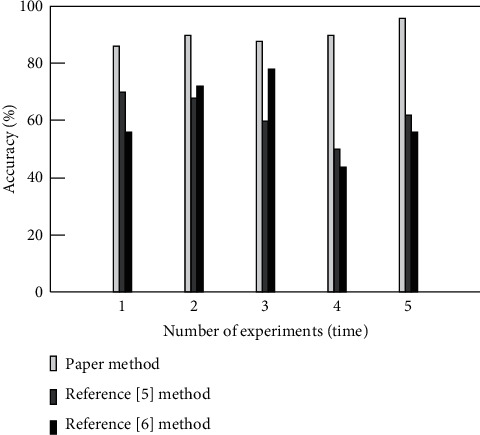
Recommendation accuracy of different methods.

In order to verify the effect of online education resource recommendation, reference [5] method, reference [6] method, and this method are used to verify the accuracy. The accuracy results of online education resource recommendation are shown in Figure 4.

As can be seen from Figure 4, compared with the traditional method, the online education resource recommendation accuracy of this method is significantly higher, always higher than 80%, and the maximum value reaches 97%. However, the online education resource recommendation accuracy of reference [5] method and reference [6] method is low. Among them, the maximum recommendation accuracy of reference [6] method is 78%, and the recommendation accuracy of reference [5] method is lower. It can be seen that this method has better recommendation effect and can provide more accurate resources for resource demanders. This is because this method uses firefly algorithm to classify online education resources and constructs vector space function to filter the classified online education resources, which effectively improves the accuracy of recommendation.

#### 3.2.2. Comparison of Online Educational Resources Recommendation Efficiency

Recommendation efficiency describes the time consumed by resource recommendation. Therefore, under the same test conditions, the methods of reference [5], reference [6], and this paper are used to count the time consumed by resource recommendation. The results are shown in Table 2.

It can be seen from Table 2 that, during the testing process, the recommended time for resources of this method, reference [5] method, and reference [6] method has been increasing. Among them, the growth rate of this method is the smallest, and the recommended time is always controlled below 5.0 s, which is far lower than the recommended time of reference [5] method and reference [6], indicating that the recommended efficiency of this method is high. According to the above analysis, this method solves the problem of low recommendation efficiency of traditional methods and can provide users with educational resources faster. This is because this method uses association rules to collect online education resources and uses firefly algorithm to classify online education resources. The correlation between knowledge points is calculated by the knowledge association theory, and the knowledge with the highest user interest is selected as the target recommendation resource to effectively improve the recommendation efficiency.

#### 3.2.3. Comparison of User Satisfaction

**Figure 5:**
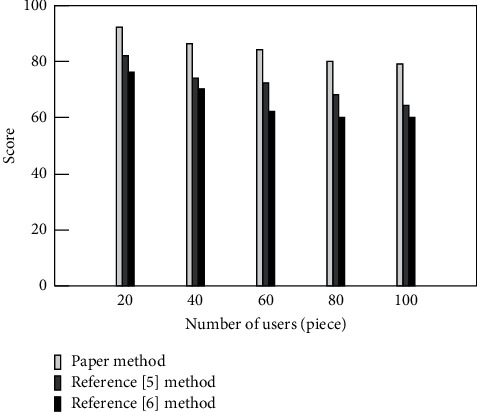
Users' satisfaction with different methods.

Online education resource recommendation is mainly for users, so users' satisfaction with the recommendation results is very important. By scoring the recommendation effect by 100 users, the users' satisfaction with the recommendation effect of the above method resources is tested. The test results are shown in Figure 5.

It can be seen from Figure 5 that, with the increase of the number of users, the average number of user scores gradually decreases. Through comparison, it can be seen that users are more satisfied with the recommendation effect of the method in this paper, and the score always remains above 80 points, [5] The satisfaction score of the method in [6] is basically below 80 points. It can be seen that users are more satisfied with the method in this paper, which also reflects the better recommendation effect of this method from the side. This is because this method uses firefly algorithm to classify online education resources. The correlation between knowledge points is calculated by the knowledge association theory, and the knowledge with the highest user interest is selected as the target recommendation resource, which effectively improves the user's satisfaction.

## 4. Conclusion

This paper proposes a personalized recommendation algorithm for online education resources based on knowledge association. Collect online education resources according to association rules, classify online education resources using firefly algorithm, filter the classified online education resources by constructing vector space function, calculate the correlation between knowledge points based on knowledge association theory, and select the knowledge with the highest user interest as the target recommendation resource according to the measurement results to realize the personalized recommendation of online education resources. The following conclusions are drawn through experiments:

1. The online educational resource recommendation accuracy of the method in this paper is significantly higher, always higher than 80%, and the maximum value can reach 97%.
2. The maximum resource recommendation accuracy rate of the method in this paper is 97%, the recommendation time is always controlled below 5.0 s, and the user's satisfaction with it is higher, indicating that its recommendation effect is better.
3. Users are more satisfied with the recommendation effect of the method in this paper, and the score is always above 80 points, which reflects the better recommendation effect of this method.

Under the method of this paper, the satisfaction of recommendation effect has been significantly improved, but the accuracy of education resources recommendation still needs to be further improved.

## Figures and Tables

**Table 1:** Dataset statistics.

Dataset	Number of learners	Number of exercises	Knowledge points
IntAddSub	753	1056	13
FrcSub	536	20	8

**Table 2:** Online educational resource recommendation efficiency of different methods.

Number of experiments/times	Online educational resource recommendation time/s		
	Reference [5] method	Reference [6] method	This paper method
1	1.6	3.1	2.9
2	2.0	4.9	3.5
3	3.1	5.2	4.7
4	3.4	6.9	5.0
5	3.5	7.1	6.3
6	4.0	8.1	7.1
7	4.2	8.9	7.5
8	4.5	9.8	8.2
9	4.8	10.2	8.8
10	4.9	11.1	9.0

## Data Availability

The data used to support the findings of this study can be obtained from the corresponding author upon request.
